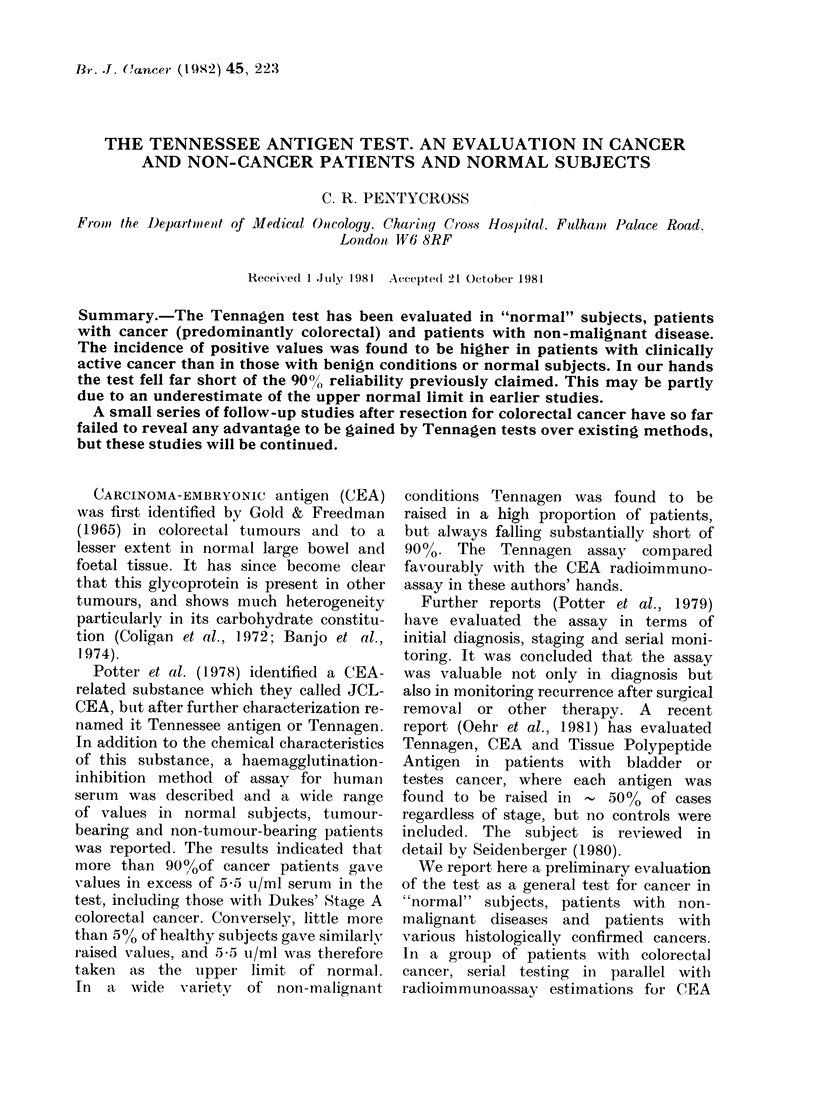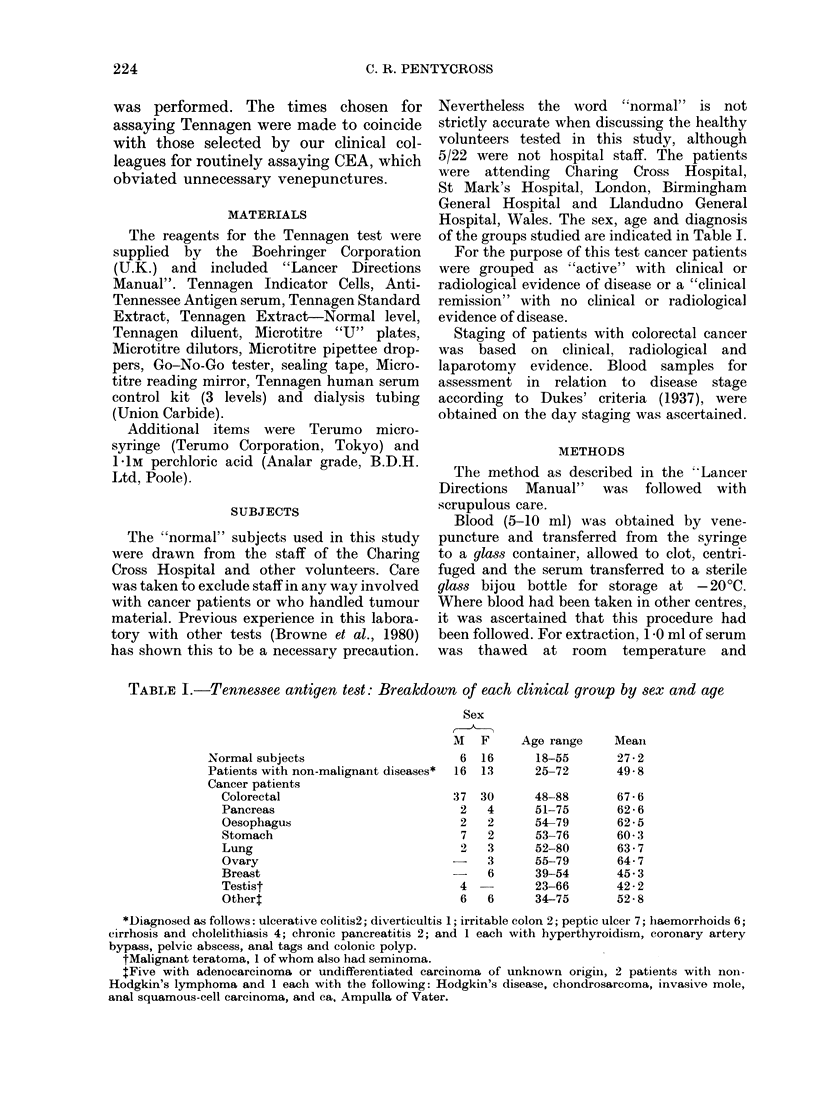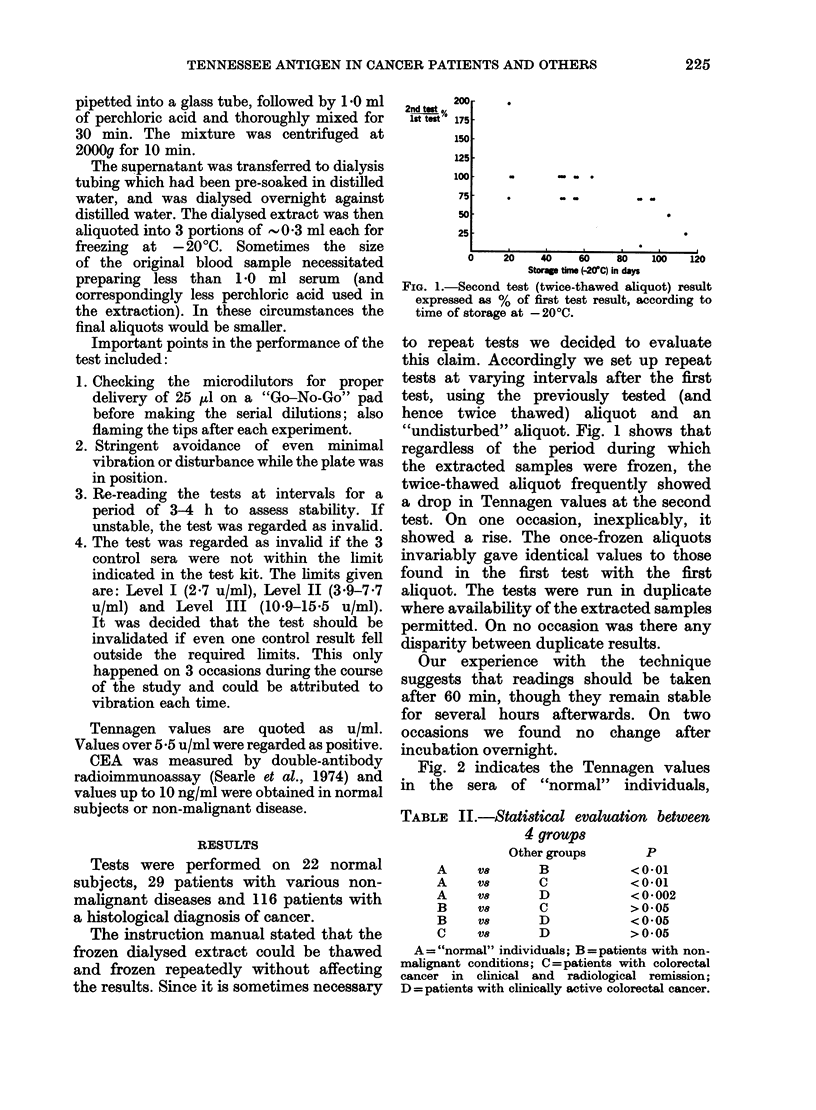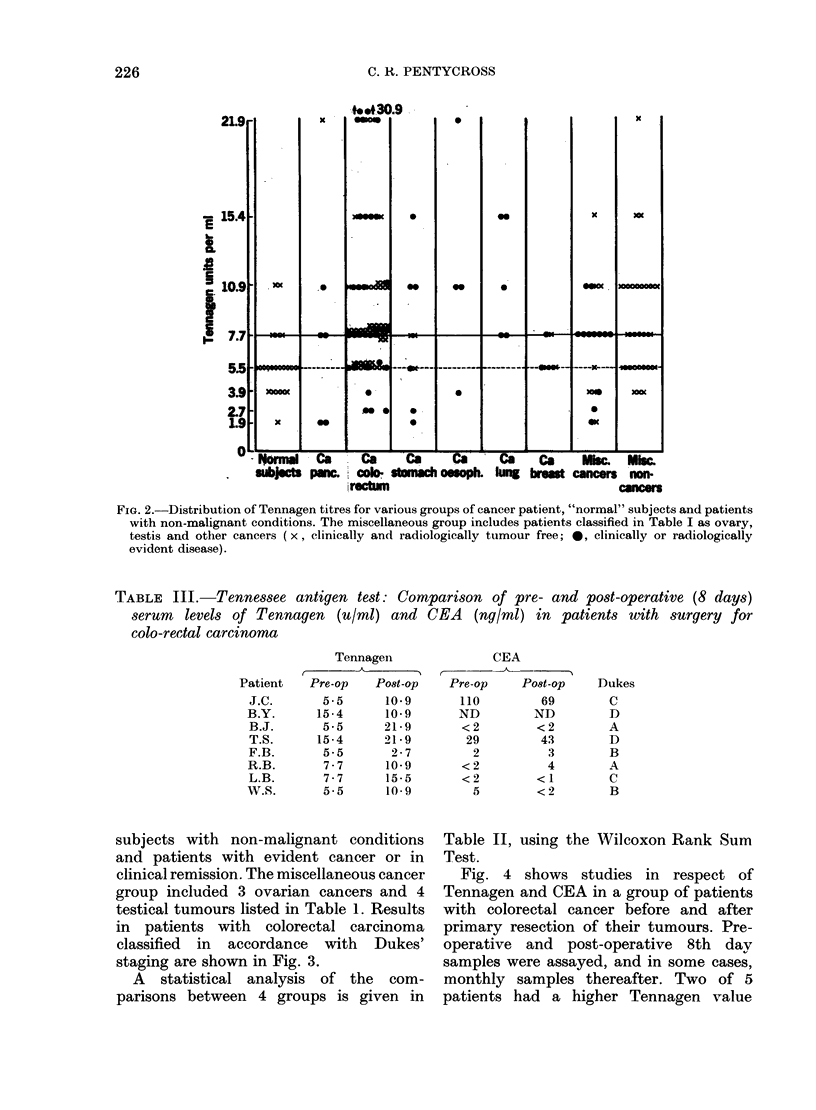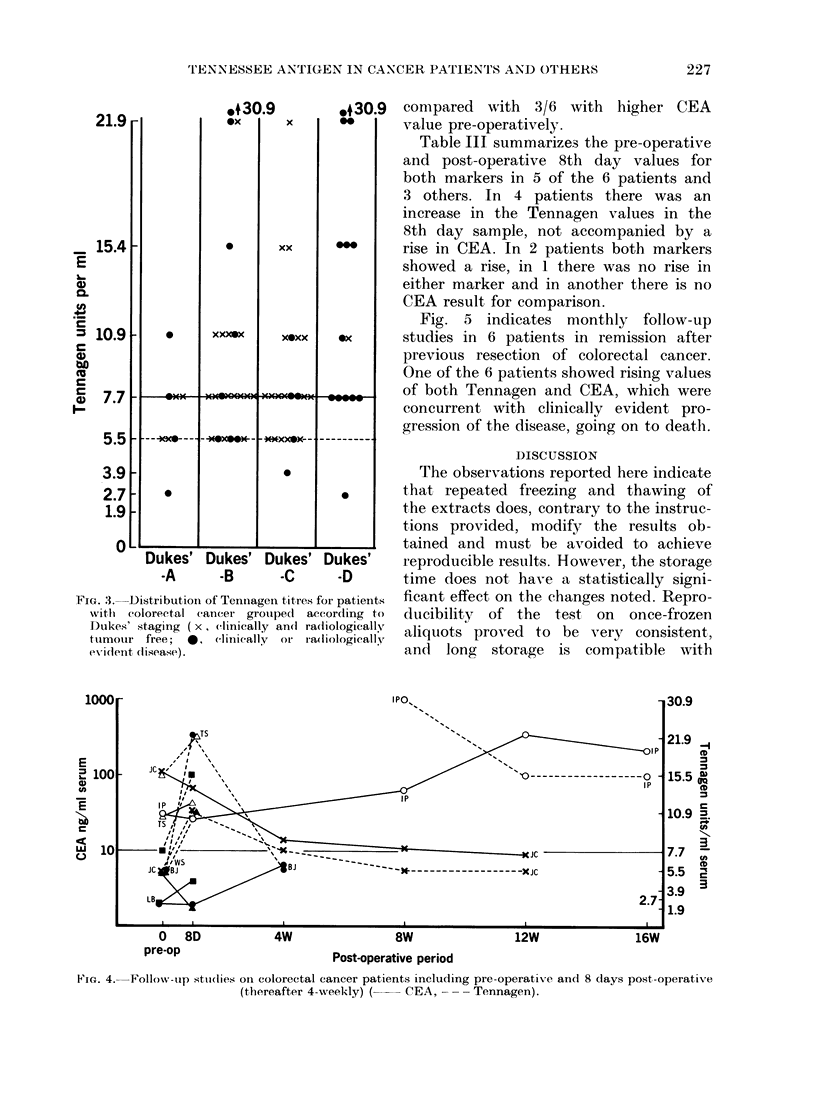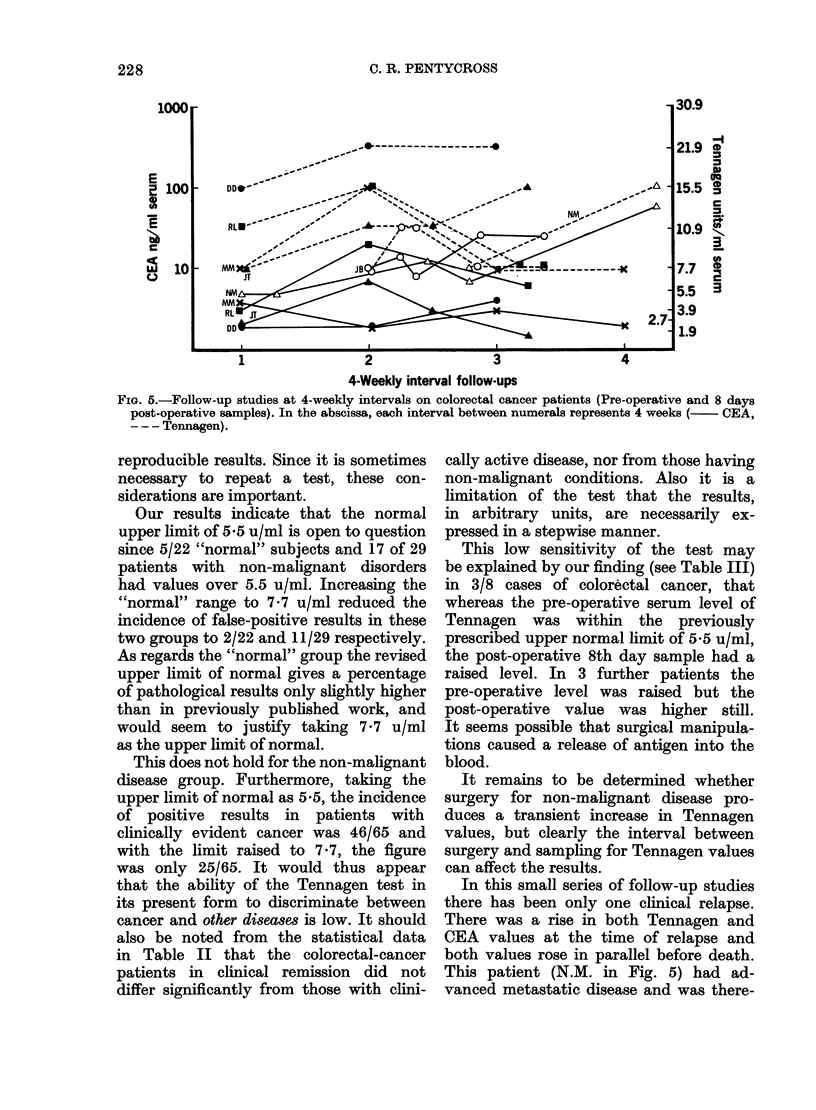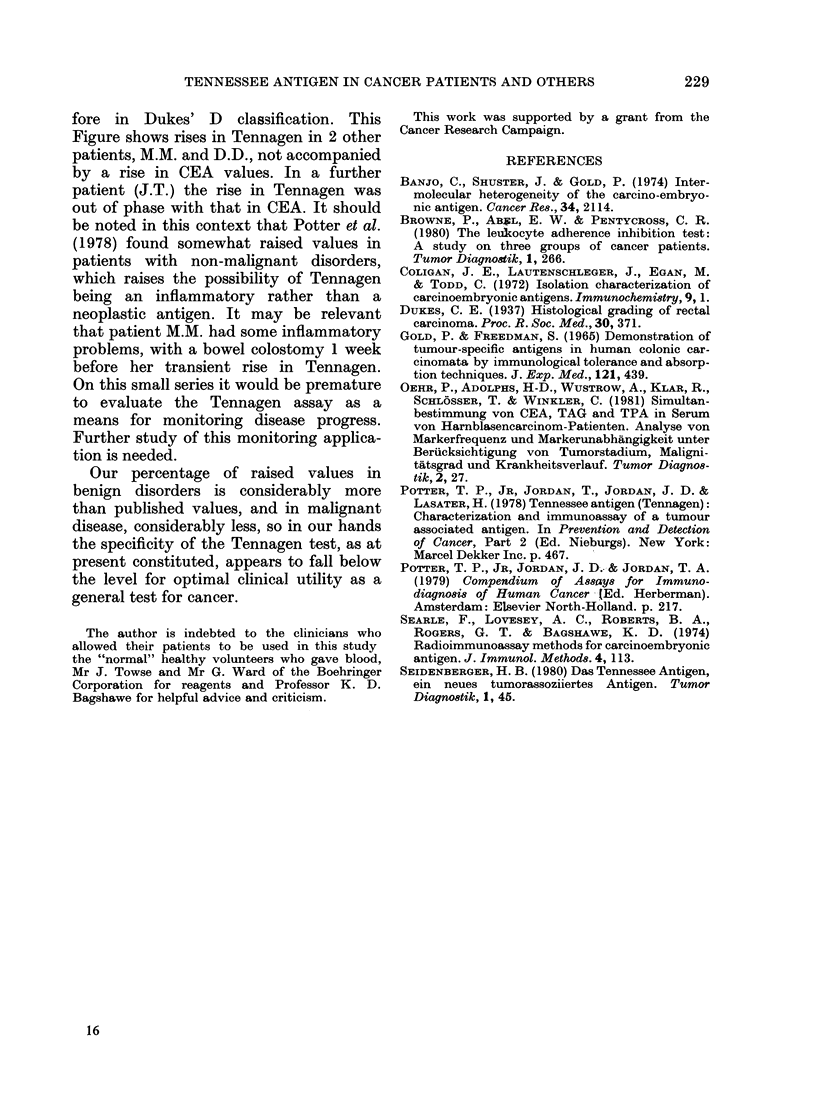# The Tennessee antigen test. An evaluation in cancer and non-cancer patients and normal subjects.

**DOI:** 10.1038/bjc.1982.37

**Published:** 1982-02

**Authors:** C. R. Pentycross

## Abstract

The Tennagen test has been evaluated in "normal" subjects, patients with cancer (predominantly colorectal) and patients with non-malignant disease. The incidence of positive values was found to be higher in patients with clinically active cancer than in those with benign conditions or normal subjects. In our hands the test fell far short of the 90% reliability previously claimed. This may be partly due to an underestimate of the upper normal limit in earlier studies. A small series of follow-up studies after resection for colorectal cancer have so far failed to reveal any advantage to be gained by Tennagen tests over existing methods, but these studied will be continued.


Br. J. Cancer (1982) 45, 223

### THE TENNESSEE ANTIGEN TEST. AN EVALUATION IN CANCER AND NON-CANCER PATIENTS AND NORMAL SUBJECTS

C. R. PENTYCROSS

*From the Department of Medical Oncology, Charing Cross Hospital, Fulham Palace Road, London W6 8RF*

Received 1 July 1981 Accepted 21 October 1981

**Summary.—The Tennagen test has been evaluated in "normal" subjects, patients with cancer (predominantly colorectal) and patients with non-malignant disease. The incidence of positive values was found to be higher in patients with clinically active cancer than in those with benign conditions or normal subjects. In our hands the test fell far short of the 90% reliability previously claimed. This may be partly due to an underestimate of the upper normal limit in earlier studies.**

**A small series of follow-up studies after resection for colorectal cancer have so far failed to reveal any advantage to be gained by Tennagen tests over existing methods, but these studies will be continued.**

CARCINOMA-EMBRYONIC antigen (CEA) was first identified by Gold & Freedman (1965) in colorectal tumours and to a lesser extent in normal large bowel and foetal tissue. It has since become clear that this glycoprotein is present in other tumours, and shows much heterogeneity particularly in its carbohydrate constitution (Coligan *et al.*, 1972; Banjo *et al.*, 1974).

Potter *et al.* (1978) identified a CEA-related substance which they called JCL-CEA, but after further characterization renamed it Tennessee antigen or Tennagen. In addition to the chemical characteristics of this substance, a haemagglutination-inhibition method of assay for human serum was described and a wide range of values in normal subjects, tumour-bearing and non-tumour-bearing patients was reported. The results indicated that more than 90%of cancer patients gave values in excess of 5·5 u/ml serum in the test, including those with Dukes' Stage A colorectal cancer. Conversely, little more than 5% of healthy subjects gave similarly raised values, and 5·5 u/ml was therefore taken as the upper limit of normal. In a wide variety of non-malignant conditions Tennagen was found to be raised in a high proportion of patients, but always falling substantially short of 90%. The Tennagen assay compared favourably with the CEA radioimmunoassay in these authors' hands.

Further reports (Potter *et al.*, 1979) have evaluated the assay in terms of initial diagnosis, staging and serial monitoring. It was concluded that the assay was valuable not only in diagnosis but also in monitoring recurrence after surgical removal or other therapy. A recent report (Oehr *et al.*, 1981) has evaluated Tennagen, CEA and Tissue Polypeptide Antigen in patients with bladder or testes cancer, where each antigen was found to be raised in ~ 50% of cases regardless of stage, but no controls were included. The subject is reviewed in detail by Seidenberger (1980).

We report here a preliminary evaluation of the test as a general test for cancer in "normal" subjects, patients with non-malignant diseases and patients with various histologically confirmed cancers. In a group of patients with colorectal cancer, serial testing in parallel with radioimmunoassay estimations for CEA

was performed. The times chosen for assaying Tennagen were made to coincide with those selected by our clinical colleagues for routinely assaying CEA, which obviated unnecessary venepunctures.

### MATERIALS

The reagents for the Tennagen test were supplied by the Boehringer Corporation (U.K.) and included "Lancer Directions Manual". Tennagen Indicator Cells, Anti-Tennessee Antigen serum, Tennagen Standard Extract, Tennagen Extract—Normal level, Tennagen diluent, Microtitre "U" plates, Microtitre dilutors, Microtitre pipettee droppers, Go-No-Go tester, sealing tape, Microtitre reading mirror, Tennagen human serum control kit (3 levels) and dialysis tubing (Union Carbide).

Additional items were Terumo microsyringe (Terumo Corporation, Tokyo) and 1·1M perchloric acid (Analar grade, B.D.H. Ltd, Poole).

### SUBJECTS

The "normal" subjects used in this study were drawn from the staff of the Charing Cross Hospital and other volunteers. Care was taken to exclude staff in any way involved with cancer patients or who handled tumour material. Previous experience in this laboratory with other tests (Browne *et al.*, 1980) has shown this to be a necessary precaution. Nevertheless the word "normal" is not strictly accurate when discussing the healthy volunteers tested in this study, although 5/22 were not hospital staff. The patients were attending Charing Cross Hospital, St Mark's Hospital, London, Birmingham General Hospital and Llandudno General Hospital, Wales. The sex, age and diagnosis of the groups studied are indicated in Table I.

For the purpose of this test cancer patients were grouped as "active" with clinical or radiological evidence of disease or a "clinical remission" with no clinical or radiological evidence of disease.

Staging of patients with colorectal cancer was based on clinical, radiological and laparotomy evidence. Blood samples for assessment in relation to disease stage according to Dukes' criteria (1937), were obtained on the day staging was ascertained.

### METHODS

The method as described in the "Lancer Directions Manual" was followed with scrupulous care.

Blood (5–10 ml) was obtained by venepuncture and transferred from the syringe to a *glass* container, allowed to clot, centrifuged and the serum transferred to a sterile *glass* bijou bottle for storage at −20°C. Where blood had been taken in other centres, it was ascertained that this procedure had been followed. For extraction, 1·0 ml of serum was thawed at room temperature and

TABLE I.—*Tennessee antigen test: Breakdown of each clinical group by sex and age*

| | Sex | | | |
|---|---|---|---|---|
| | M | F | Age range | Mean |
| Normal subjects | 6 | 16 | 18–55 | 27·2 |
| Patients with non-malignant diseases* | 16 | 13 | 25–72 | 49·8 |
| Cancer patients | | | | |
| Colorectal | 37 | 30 | 48–88 | 67·6 |
| Pancreas | 2 | 4 | 51–75 | 62·6 |
| Oesophagus | 2 | 2 | 54–79 | 62·5 |
| Stomach | 7 | 2 | 53–76 | 60·3 |
| Lung | 2 | 3 | 52–80 | 63·7 |
| Ovary | — | 3 | 55–79 | 64·7 |
| Breast | — | 6 | 39–54 | 45·3 |
| Testis† | 4 | — | 23–66 | 42·2 |
| Other‡ | 6 | 6 | 34–75 | 52·8 |

*Diagnosed as follows: ulcerative colitis2; diverticultis 1; irritable colon 2; peptic ulcer 7; haemorrhoids 6; cirrhosis and cholelithiasis 4; chronic pancreatitis 2; and 1 each with hyperthyroidism, coronary artery bypass, pelvic abscess, anal tags and colonic polyp.

†Malignant teratoma, 1 of whom also had seminoma.

‡Five with adenocarcinoma or undifferentiated carcinoma of unknown origin, 2 patients with non-Hodgkin's lymphoma and 1 each with the following: Hodgkin's disease, chondrosarcoma, invasive mole, anal squamous-cell carcinoma, and ca. Ampulla of Vater.

pipetted into a glass tube, followed by 1·0 ml of perchloric acid and thoroughly mixed for 30 min. The mixture was centrifuged at 2000*g* for 10 min.

The supernatant was transferred to dialysis tubing which had been pre-soaked in distilled water, and was dialysed overnight against distilled water. The dialysed extract was then aliquoted into 3 portions of ~0·3 ml each for freezing at −20°C. Sometimes the size of the original blood sample necessitated preparing less than 1·0 ml serum (and correspondingly less perchloric acid used in the extraction). In these circumstances the final aliquots would be smaller.

Important points in the performance of the test included:

1. Checking the microdilutors for proper delivery of 25 μl on a "Go–No-Go" pad before making the serial dilutions; also flaming the tips after each experiment.
2. Stringent avoidance of even minimal vibration or disturbance while the plate was in position.
3. Re-reading the tests at intervals for a period of 3–4 h to assess stability. If unstable, the test was regarded as invalid.
4. The test was regarded as invalid if the 3 control sera were not within the limit indicated in the test kit. The limits given are: Level I (2·7 u/ml), Level II (3·9–7·7 u/ml) and Level III (10·9–15·5 u/ml). It was decided that the test should be invalidated if even one control result fell outside the required limits. This only happened on 3 occasions during the course of the study and could be attributed to vibration each time.

Tennagen values are quoted as u/ml. Values over 5·5 u/ml were regarded as positive.

CEA was measured by double-antibody radioimmunoassay (Searle *et al.*, 1974) and values up to 10 ng/ml were obtained in normal subjects or non-malignant disease.

### RESULTS

Tests were performed on 22 normal subjects, 29 patients with various non-malignant diseases and 116 patients with a histological diagnosis of cancer.

The instruction manual stated that the frozen dialysed extract could be thawed and frozen repeatedly without affecting the results. Since it is sometimes necessary

Fig. 1.—Second test (twice-thawed aliquot) result expressed as % of first test result, according to time of storage at −20°C.

to repeat tests we decided to evaluate this claim. Accordingly we set up repeat tests at varying intervals after the first test, using the previously tested (and hence twice thawed) aliquot and an "undisturbed" aliquot. Fig. 1 shows that regardless of the period during which the extracted samples were frozen, the twice-thawed aliquot frequently showed a drop in Tennagen values at the second test. On one occasion, inexplicably, it showed a rise. The once-frozen aliquots invariably gave identical values to those found in the first test with the first aliquot. The tests were run in duplicate where availability of the extracted samples permitted. On no occasion was there any disparity between duplicate results.

Our experience with the technique suggests that readings should be taken after 60 min, though they remain stable for several hours afterwards. On two occasions we found no change after incubation overnight.

Fig. 2 indicates the Tennagen values in the sera of "normal" individuals,

Table II.—*Statistical evaluation between 4 groups*

| | | Other groups | *P* |
|---|---|---|---|
| A | *vs* | B | <0·01 |
| A | *vs* | C | <0·01 |
| A | *vs* | D | <0·002 |
| B | *vs* | C | >0·05 |
| B | *vs* | D | <0·05 |
| C | *vs* | D | >0·05 |

A="normal" individuals; B=patients with non-malignant conditions; C=patients with colorectal cancer in clinical and radiological remission; D=patients with clinically active colorectal cancer.

FIG. 2.—Distribution of Tennagen titres for various groups of cancer patient, "normal" subjects and patients with non-malignant conditions. The miscellaneous group includes patients classified in Table I as ovary, testis and other cancers (×, clinically and radiologically tumour free; ●, clinically or radiologically evident disease).

TABLE III.—*Tennessee antigen test: Comparison of pre- and post-operative (8 days) serum levels of Tennagen (u/ml) and CEA (ng/ml) in patients with surgery for colo-rectal carcinoma*

| | Tennagen | | CEA | | |
|---|---|---|---|---|---|
| Patient | *Pre-op* | *Post-op* | *Pre-op* | *Post-op* | Dukes |
| J.C. | 5·5 | 10·9 | 110 | 69 | C |
| B.Y. | 15·4 | 10·9 | ND | ND | D |
| B.J. | 5·5 | 21·9 | < 2 | < 2 | A |
| T.S. | 15·4 | 21·9 | 29 | 43 | D |
| F.B. | 5·5 | 2·7 | 2 | 3 | B |
| R.B. | 7·7 | 10·9 | < 2 | 4 | A |
| L.B. | 7·7 | 15·5 | < 2 | < 1 | C |
| W.S. | 5·5 | 10·9 | 5 | < 2 | B |

subjects with non-malignant conditions and patients with evident cancer or in clinical remission. The miscellaneous cancer group included 3 ovarian cancers and 4 testical tumours listed in Table 1. Results in patients with colorectal carcinoma classified in accordance with Dukes' staging are shown in Fig. 3.

A statistical analysis of the comparisons between 4 groups is given in Table II, using the Wilcoxon Rank Sum Test.

Fig. 4 shows studies in respect of Tennagen and CEA in a group of patients with colorectal cancer before and after primary resection of their tumours. Pre-operative and post-operative 8th day samples were assayed, and in some cases, monthly samples thereafter. Two of 5 patients had a higher Tennagen value

FIG. 3.—Distribution of Tennagen titres for patients with colorectal cancer grouped according to Dukes' staging (×, clinically and radiologically tumour free; ●, clinically or radiologically evident disease).

compared with 3/6 with higher CEA value pre-operatively.

Table III summarizes the pre-operative and post-operative 8th day values for both markers in 5 of the 6 patients and 3 others. In 4 patients there was an increase in the Tennagen values in the 8th day sample, not accompanied by a rise in CEA. In 2 patients both markers showed a rise, in 1 there was no rise in either marker and in another there is no CEA result for comparison.

Fig. 5 indicates monthly follow-up studies in 6 patients in remission after previous resection of colorectal cancer. One of the 6 patients showed rising values of both Tennagen and CEA, which were concurrent with clinically evident progression of the disease, going on to death.

DISCUSSION

The observations reported here indicate that repeated freezing and thawing of the extracts does, contrary to the instructions provided, modify the results obtained and must be avoided to achieve reproducible results. However, the storage time does not have a statistically significant effect on the changes noted. Reproducibility of the test on once-frozen aliquots proved to be very consistent, and long storage is compatible with

FIG. 4.—Follow-up studies on colorectal cancer patients including pre-operative and 8 days post-operative (thereafter 4-weekly) (—— CEA, – – – Tennagen).

FIG. 5.—Follow-up studies at 4-weekly intervals on colorectal cancer patients (Pre-operative and 8 days post-operative samples). In the abscissa, each interval between numerals represents 4 weeks (—— CEA, – – – Tennagen).

reproducible results. Since it is sometimes necessary to repeat a test, these considerations are important.

Our results indicate that the normal upper limit of 5·5 u/ml is open to question since 5/22 "normal" subjects and 17 of 29 patients with non-malignant disorders had values over 5.5 u/ml. Increasing the "normal" range to 7·7 u/ml reduced the incidence of false-positive results in these two groups to 2/22 and 11/29 respectively. As regards the "normal" group the revised upper limit of normal gives a percentage of pathological results only slightly higher than in previously published work, and would seem to justify taking 7·7 u/ml as the upper limit of normal.

This does not hold for the non-malignant disease group. Furthermore, taking the upper limit of normal as 5·5, the incidence of positive results in patients with clinically evident cancer was 46/65 and with the limit raised to 7·7, the figure was only 25/65. It would thus appear that the ability of the Tennagen test in its present form to discriminate between cancer and *other diseases* is low. It should also be noted from the statistical data in Table II that the colorectal-cancer patients in clinical remission did not differ significantly from those with clinically active disease, nor from those having non-malignant conditions. Also it is a limitation of the test that the results, in arbitrary units, are necessarily expressed in a stepwise manner.

This low sensitivity of the test may be explained by our finding (see Table III) in 3/8 cases of colorectal cancer, that whereas the pre-operative serum level of Tennagen was within the previously prescribed upper normal limit of 5·5 u/ml, the post-operative 8th day sample had a raised level. In 3 further patients the pre-operative level was raised but the post-operative value was higher still. It seems possible that surgical manipulations caused a release of antigen into the blood.

It remains to be determined whether surgery for non-malignant disease produces a transient increase in Tennagen values, but clearly the interval between surgery and sampling for Tennagen values can affect the results.

In this small series of follow-up studies there has been only one clinical relapse. There was a rise in both Tennagen and CEA values at the time of relapse and both values rose in parallel before death. This patient (N.M. in Fig. 5) had advanced metastatic disease and was therefore in Dukes' D classification. This Figure shows rises in Tennagen in 2 other patients, M.M. and D.D., not accompanied by a rise in CEA values. In a further patient (J.T.) the rise in Tennagen was out of phase with that in CEA. It should be noted in this context that Potter *et al.* (1978) found somewhat raised values in patients with non-malignant disorders, which raises the possibility of Tennagen being an inflammatory rather than a neoplastic antigen. It may be relevant that patient M.M. had some inflammatory problems, with a bowel colostomy 1 week before her transient rise in Tennagen. On this small series it would be premature to evaluate the Tennagen assay as a means for monitoring disease progress. Further study of this monitoring application is needed.

Our percentage of raised values in benign disorders is considerably more than published values, and in malignant disease, considerably less, so in our hands the specificity of the Tennagen test, as at present constituted, appears to fall below the level for optimal clinical utility as a general test for cancer.

The author is indebted to the clinicians who allowed their patients to be used in this study the "normal" healthy volunteers who gave blood, Mr J. Towse and Mr G. Ward of the Boehringer Corporation for reagents and Professor K. D. Bagshawe for helpful advice and criticism.

This work was supported by a grant from the Cancer Research Campaign.

### REFERENCES

BANJO, C., SHUSTER, J. & GOLD, P. (1974) Intermolecular heterogeneity of the carcino-embryonic antigen. *Cancer Res.*, **34**, 2114.

BROWNE, P., ABEL, E. W. & PENTYCROSS, C. R. (1980) The leukocyte adherence inhibition test: A study on three groups of cancer patients. *Tumor Diagnostik*, **1**, 266.

COLIGAN, J. E., LAUTENSCHLEGER, J., EGAN, M. & TODD, C. (1972) Isolation characterization of carcinoembryonic antigens. *Immunochemistry*, **9**, 1.

DUKES, C. E. (1937) Histological grading of rectal carcinoma. *Proc. R. Soc. Med.*, **30**, 371.

GOLD, P. & FREEDMAN, S. (1965) Demonstration of tumour-specific antigens in human colonic carcinomata by immunological tolerance and absorption techniques. J. *Exp. Med.*, **121**, 439.

OEHR, P., ADOLPHS, H-D., WUSTROW, A., KLAR, R., SCHLÖSSER, T. & WINKLER, C. (1981) Simultanbestimmung von CEA, TAG and TPA in Serum von Harnblasencarcinom-Patienten. Analyse von Markerfrequenz und Markerunabhängigkeit unter Berücksichtigung von Tumorstadium, Malignitätsgrad und Krankheitsverlauf. *Tumor Diagnostik*, **2**, 27.

POTTER, T. P., JR, JORDAN, T., JORDAN, J. D. & LASATER, H. (1978) Tennessee antigen (Tennagen): Characterization and immunoassay of a tumour associated antigen. In *Prevention and Detection of Cancer*, Part 2 (Ed. Nieburgs). New York: Marcel Dekker Inc. p. 467.

POTTER, T. P., JR, JORDAN, J. D. & JORDAN, T. A. (1979) *Compendium of Assays for Immunodiagnosis of Human Cancer* (Ed. Herberman). Amsterdam: Elsevier North-Holland. p. 217.

SEARLE, F., LOVESEY, A. C., ROBERTS, B. A., ROGERS, G. T. & BAGSHAWE, K. D. (1974) Radioimmunoassay methods for carcinoembryonic antigen. *J. Immunol. Methods*. **4**, 113.

SEIDENBERGER, H. B. (1980) Das Tennessee Antigen, ein neues tumorassoziiertes Antigen. *Tumor Diagnostik*, **1**, 45.